# Applications of sensory and physiological measurement in oral‐facial dental pain

**DOI:** 10.1111/scd.12323

**Published:** 2018-09-08

**Authors:** Darya Dabiri, Daniel E. Harper, Yvonne Kapila, Grant H. Kruger, Daniel J. Clauw, Steven Harte

**Affiliations:** ^1^ Chronic Pain & Fatigue Research Center Department of Anesthesiology University of Michigan Medicine Ann Arbor Michigan; ^2^ Division of Periodontology, Department of Orofacial Sciences University of California San Francisco San Francisco California; ^3^ Department of Mechanical Engineering University of Michigan Ann Arbor Michigan

**Keywords:** cognitive impairment, dental pain, orafacial pain, quantitative sensory testing

## Abstract

Dentists regularly employ a variety of self‐report and sensory techniques to aid in the diagnosis and treatment of tooth‐related disease. Many of these techniques leverage principles borrowed from psychophysics, the quantitative measurement of the relationship between stimuli and evoked sensations, which falls under the larger umbrella of quantitative sensory testing (QST). However, most clinicians fail to meet the bar for what could be considered quantitative sensory testing, and instead focus on qualitative and dichotomous “yes/no” aspects of sensory experience. With our current subjective measurements for pain assessments, diagnosis and treatment of dental pain in young children and individuals (any age) with severe cognitive impairment rely extensively on third‐party observations. Consequently, the limitation of inadequate pain diagnosis can lead to poor pain management. In this review, it discusses mechanisms that underlie acute and chronic dental pain. It details the measurement of somatosensory responses and pulpal blood flow as objective measures of tooth health and pain. It proposes that bridging these varied methodologies will significantly improve diagnosis and treatment of orofacial pain and pathology. It concludes that improving the precision of sensory measurements could yield important improvements in diagnostic challenges in pulpal pathology for noncommunicative and cognitively impaired individuals.

## 1 INTRODUCTION

Pain motivates individuals to seek dental care.^1^ The perception of pain is a complex process that involves bidirectional communication between the central and peripheral nervous systems. It is now known that individuals vary widely in their pain sensitivity, and there often is a very poor relationship between the degree of peripheral damage/inflammation within an individual and how much pain he/she is experiencing. This is especially true in subacute or chronic pain conditions, where frequently there is little evidence of ongoing damage or inflammation in the periphery.^2^ In these cases, intervening in the periphery – as dentists are trained to do – will not alleviate pain, and could actually worsen a person's clinical condition. Therefore, it is critically important that the field of dentistry progresses toward a mechanistic understanding of pain to better identify individuals who are at risk of failing to respond to our interventions and to limit procedures to only those who are likely to benefit from them.

Traditionally, dentists and other oral health clinicians have used relatively crude “chairside” tests to infer whether there is underlying pathology in oral structures. Typical intraoral examinations include palpation, percussion, thermal, electric, and periodontal examinations. While valuable, these methods often lack quantifiable and objective outcomes, and there remains considerable inter‐ and intraclinician variation in their implementation. In addition, determination of pain and pathology in young children, adults with a language barrier, or individuals of any age with cognitive impairment (CI) is very challenging. In contrast to these subjective measures, standardized tests of perceptual and physiological reactions to externally applied and quantifiable stimuli (i.e., quantitative sensory testing – QST) mitigates these limitations and provides a more accurate approach to identify pain mechanisms and track changes in sensory function over time. In this review, we provide an overview of QST methods and discuss how they can be implemented in standard dental practice. The “Mechanisms of Orofacial Pain” section reviews pain mechanisms that contribute to acute and chronic dental pain. The “QST in Orofacial Pain and Dentistry” section discusses various physiological signals that can be monitored to determine tooth health and pulpal vitality. The “Noncommunicative Patients and Challenges in Dental Pain Assessments” section describes the use of QST procedures in the study orofacial pain. The “Bridging the Gap between QST and Pulp Vitality Testing to Improve Clinical Care” section describes challenges in the diagnosis of dental pain in noncommunicative individuals. Finally, the “Conclusion” section provides recommendations on how these sensory and physiological measurement technologies can be combined to improve clinical dental care.

## 2 MECHANISMS OF OROFACIAL PAIN

### 2.1 Nociceptive pain

Nociceptive pain is the most common form of pain in the orofacial region and normally occurs following acute stimulation of the nociceptors embedded in the skin, intraoral cavity, and dental pulp. Inflammatory pain is also categorized under this umbrella term, since inflammation in the periphery is known to sensitize nociceptors and increase their spontaneous firing rate and their excitability to stimulation. Clinical pain is a reflection of the nociceptive circuits’ overall excitability, and not just a pain system being “turned on” by a pathology. The sensitivity of those nociceptive circuits can be shifted and changed by innocuous stimuli, more like a dial than a switch, and the state of excitability can dictate the level of pain experienced.^3^, ^4^

The majority of tooth pain is thought to be nociceptive and of odontogenic origin.^5^ Odontogenic pain encompasses pain that could originate from either pulpal or periodontal tissue (mucosa, gingiva, or periodontal ligament).^6^ Orofacial nociceptive pain often originates from insult of the dental pulp. The rigid compartment that pulp resides in provides a support structure and protects it from the microbes present in the mouth. When that protective chamber is damaged or corroded, the pulp it encapsulates becomes susceptible to the hostile elements present in the oral cavity. The microcirculation within the healthy pulp plays a crucial role in orchestrating inflammatory response in response to pulpal damages.^7^, ^8^ Inflammation of the pulp, or pulpitis, produces increasingly intense and prolonged painful responses to thermal or osmotic stimulation, as distinct from the less painful and phasic responses seen with normal dentine sensitivity. Early stage inflammatory responses may be reversible, but as the pathology advances, the process becomes irreversible, and may result in the development of spontaneous pain that occurs without provocation, likely because of both peripheral and central sensitization. Chronic inflammation can eventually lead to pulpal necrosis and periapical pathology.^9^

Pulpitis can be either reversible or irreversible. Reversible pulpitis is often characterized by a brief, sharp nonspontaneous pain upon provocation.^10^ This transient pulpal inflammation can be reverted once the source of irritation is removed (e.g., caries or occlusal trauma). In contrast, irreversible pulpitis is characterized by pain that lingers following stimulation and spontaneous pain that occurs without provocation. Treatment for irreversible pulpitis involves either excavation of the diseased pulp or tooth extraction. Irreversible pulpitis, left untreated, leads to necrosis – a necrotic pulp will usually not respond to a thermal or electrical stimulation. Teeth with irreversible pulpitis can be completely asymptomatic or extremely painful on percussion,^10^, ^11^ which limits the usefulness of this technique in diagnosis. Symptoms of odontogenic pain can vary greatly, in some cases pulpal necrosis occurs without any prior symptomatic pulpitis.^12^ Studies have shown that clinical pain symptoms do not necessarily correlate with histological findings in pulp.^13^, ^14^, ^15^

Orofacial nociceptive pain could also originate from temporomandibular joint (TMJ) structures. There are at least three potential etiologies for TMD pain including degradation of TMJ structures, inflammation in the joint (i.e., degenerative joint disease‐arthritis) and myofascial pain. There is limited evidence that peripheral inflammation in local musculature (e.g., temporalis, medial pterygoid, and masseter) contributes to myalgic pain in TMD,^16^ and instead this type of TMD pain is thought to occur mainly via the central nervous system (CNS) mechanisms.^17^, ^18^, ^19^

In the otherwise healthy individual, acute dental pain is often sudden and debilitating. Despite its unexpected and concerning nature, it has a protective effect as its intensity mirrors the amount of injury to the tissue and its presence motivates behaviors that aid recuperation and healing.^9^, ^20^ However, in patients with chronic or subacute pain conditions, pain signals can be augmented or amplified by the CNS, and pain often occurs either disproportionate to or in the absence of ongoing nociceptive input. Both neuropathic and centralized pain mechanisms, discussed below, are hallmark features of chronic pain.

### 2.2 Neuropathic pain

Neuropathic pain occurs following destruction to or compression of the peripheral nerves that transmit somatosensory signals to the CNS. It has typical clinical features regardless of where in the body it occurs that aid in its diagnosis. For instance, neuropathic pain is often characterized as waxing and waning, and lancinating, and it may be accompanied by paresthesias, numbness, tingling, and shooting sensations.^21^, ^22^, ^23^, ^24^, ^25^, ^26^ Neuropathic pain also typically follows the distribution of one or more sensory nerves that are damaged or inflamed.

There are specific oral conditions with prominent neuropathic components. Neuropathic pain can be classified according to its location and frequency: unilateral continuous, unilateral episodic, and bilateral continuous.^19^, ^26^, ^28^ Episodic pain typically is of short duration, and produces very sharp or electric‐like sensations. Episodic neuralgias include of trigeminal and glossopharyngeal neuralgia — named after the nerve affected. Neuropathic pain in these conditions is typically episodic and unilateral, but it can be bilateral. If bilateral, multiple sclerosis is often suspected as an etiological factor, especially in younger age groups.

The continuous neuropathic pain disorders can be spontaneous or have a trigger zone and are characterized more by a burning‐type sensation. The continuous neuralgias are considered a form of deafferentation pain and can be due to trauma including surgery or metabolic disorders such as diabetic neuropathy. Examples of unilateral continuous neuropathic pain in the orofacial region include atypical odontalgia and burning mouth syndrome. Each of these is described below.

#### 2.2.1 Trigeminal neuralgia (TN) and glossopharyngeal neuralgia

TN and glossopharyngeal are defined by unilateral episodic pain that follows the distribution of one or more defined nerve pathways. TN has typical clinical features but is frequently of unknown etiology. TN is classified into Classical and Symptomatic subtypes.^19^, ^26^ Classical TN typically manifests as sudden, sharp, shooting, shock‐like pain, elicited by slight touching of “trigger points,” that can radiate. Symptomatic TN manifests with paroxysmal painful attacks of short duration affecting one or more branches of the trigeminal nerves. Etiology of the pain source varies in classical versus symptomatic TN. Unlike symptomatic TN, vascular compression is the primary cause of classical TN pain. Trigeminal postherpetic neuralgia (PHN) is an example of symptomatic TN. PHN is defined as the persistence of pain following disappearance of the rash that can last between 1 and 6 months in a herpes zoster virus infection. Sensory changes are sometimes also observed during clinical testing of PHN, including hyperalgesia and/or allodynia. Although some cases present with a clear history of nerve damage (e.g., due to dental procedure or other insult), the actual cause of the neuropathy often remains unknown and secondary causes such as autoimmune, malignancy, or infection could be typically considered.^22^, ^27^, ^34^

#### 2.2.2 Atypical odontalgia (posttraumatic trigeminal)

Atypical odontalgia or Persistent Dentoalveolar Pain Disorder (PDAP) is increasingly recognized as being caused not only by trauma to the facial skeleton, but also by various dental procedures including root canal therapy, extractions, and/or dental implants.^35^ Pain in atypical odontalgia is very clearly localized to the dentoalveolar region with or without the presence of dentition.^35^, ^36^ Pain can present as throbbing and continuous, and at times sharp. It is often provoked by light touch. History of dental treatment does not affect the onset of the disease. The source of pain is not easily recognized, and the pain can seemingly occur without any reason. This often leads to more and more unnecessary dental procedures that fail to relieve the pain.^35^, ^36^, ^37^

#### 2.2.3 Burning mouth syndrome

Burning mouth syndrome is an example of bilateral continuous neuropathic pain.^38^ It presents as a burning sensation of the intraoral soft tissue with no apparent etiology. There have been several studies suggesting various precipitating factors, but these studies show no consensus and the quality of prospective studies and case reports is lacking.^39^ The symptoms can be continuous but the intensity does vary throughout the day. Several local and systemic causes need to be excluded in diagnosing burning mouth syndrome. Local causes include candidiasis, lichen planus, herpetic infection, and xerostomia, and systemic causes include use of specific medications (e.g., angiotensin‐converting enzyme (ACE) inhibitor for hypertension therapy), hematological causes, nutritional deficiencies, and Sjogren's syndrome.

### 2.4 Centralized pain

“Central pain,” as originally described, referred to chronic pain that occurred as a result of damage to the CNS, such as thalamic pain syndrome following cerebral ischemia.^2^ Later, new terminology was introduced to describe the amplification and/or maintenance of pain by CNS mechanisms, irrespective of peripheral nociceptive input or structural damage. Terms that have been used include, “central sensitization,” “centralized pain,” “central hyper‐excitability,” and others.^40^, ^41^, ^42^, ^43^ Consensus within the pain field on this new terminology has yet to be reached. In this review, we will use the terms “central sensitization” for all molecular, structural, and functional CNS (brain and spinal) mechanisms related to pain and sensory amplification, and “centralized pain” to refer broadly to clinical phenotypes preferentially characterized by underlying mechanisms of central sensitization.^4^, ^42^, ^44^, ^45^

The clinical features of centralized pain differ from those of nociceptive or neuropathic pain. Using a pain diagram can be very helpful in diagnosing any pain patient, but it can be especially helpful in identifying neuropathic pain that follows the distribution of a peripheral nerve, or the more widespread or diffuse pain distribution that occurs with centralized pain states. The “widespreadedness” of an individual's pain often reflects the degree to which their pain has been centralized. Centralized pain can manifest anywhere in the body and in any type of tissue, and is generally more diffuse than nociceptive or neuropathic pain since many of the central gain controls for incoming nociceptive signals that are recognized to be dysregulated in centralized pain patients can affect pain signals from throughout the body, e.g., diffuse noxious inhibitory controls (DNICs).^46^ The degree to which pain can be well localized by the patient (centralized pain cannot be as well localized) is another distinguishing factor between nociceptive and centralized pain.^2^ Other clinical features of centralized pain include hypersensitivity to a variety of painful (e.g., heat, cold, electrical, pressure) and innocuous sensory stimuli (e.g., bright lights, noises, odors), and a myriad of cooccurring CNS‐organized symptoms (e.g., fatigue, sleep difficulties, mood, and memory problems).

*Referred odontogenic pain* is a type of centralized pain condition. In odontogenic pain, central sensitization begins from peripheral tissue injury at the site of tooth and supporting periodontium. At this site, inflammation modulates activation of afferent nociceptive nerve endings by lowering their firing threshold.^47^ Prolonged activation of nociceptive input to second‐order neurons facilitates augmentation of nociceptive impulses to higher brain centers. This central sensitization can manifest as secondary hyperalgesia and referred pain. Secondary hyperalgesia denotes a change to CNS that causes an augmented reaction to painful stimuli in surrounding tissue, such as the gingiva or skin.^26^ Referred pain occurs when pain manifests at a locations remote from its source.^48^

### 2.5 Investigating intraoral pathology using physiological measures

#### 2.5.1 Clinical assessment of dental pain

Since the complaint of “tooth pain” could have both odontogenic and nonodontogenic etiologies, it is crucial to conduct a comprehensive dental evaluation, including a detailed history of the present illness, and appropriate radiographic imaging. The most precise technique to assess pulp status is by histological examination of pulpal inflammation. Unfortunately, this method cannot be practiced in clinical care as it requires surgical removal of the enamel and dentin to access the pulp; hence, clinicians need to use other noninvasive metrics, such as stimulus testing, to provide additional diagnostic information for pulpal diagnosis. In these tests, teeth and surrounding structures are then evaluated via various sensation‐evoking methods including thermal (hot and cold sensitivity), electrical, percussion, and palpation testing (see the “Noncommunicative Patients and Challenges in Dental Pain Assessments” section). Interpretation of these tests requires experience, training, and knowledge of various test limitations and patient responses. Ideally, a diagnostic test will always be positive when pathology is present, and negative when pathology is absent – however, sensitivity and specificity analyses have revealed that the most common pulp tests (i.e., cold and electrical pain threshold [EPT] testing) are imperfect diagnostic tools. Most of the time, these diagnostic tests show high negative predictive values (80% to 90%) and lower positive predicted values (30% to 70%).^49^ This wide range could be due to the difficulty in predicting histological states from diagnostic tests. An earlier study suggested that there was “no reliable” association between pulp status and histological assessment.^50^

Obtaining a detailed characterization of a patient's pain is one of the most useful diagnostic strategies for dental pain, since pulpal pathology follows a sequence of changes and symptoms that vary over time.^51^ In 2005, Pau and his group proposed a validated dental pain screening questionnaire to assess patients with odontogenic pain called the Dental Pain Questionnaire (DePaQ). It consists of 14 items that assess location, frequency, and intensity of pain in the orofacial region. It also asks questions regarding tooth‐specific signs and symptoms including temperature sensitivity, biting sensitivity, and the combination of both. The DePaQ was originally designed to differentiate between three groups of odontogenic pain: (1) irreversible pulpitis/acute apical periodontitis, (2) reversible pulpitis/dentin hypersensitivity, and (3) pericoronitis. The DePaQ showed acceptable sensitivity of 0.85; however, its specificity varies across studies.^51^, ^52^ In 2017, Nixdorf stated that the results of DePaQ showed an unacceptable specificity of 0.11, which was substantially smaller than that identified in the original validation studies (i.e., 0.83).^17^

### 2.6 Measurement of somatosensory responses

In an effort to obtain more objective measures of pupal status, many investigators have examined physiological responses to stimuli applied to teeth and surrounding tissue. These tests have a long history in the study of dental pain and yet they remain poorly understood. Somatosensory responses in the orofacial tissue come from various low‐threshold somatosensory receptors originating from structures such as teeth, TMJ, skin, and muscles. These structures are mainly innervated by branches of the trigeminal (V) nerve. Oral sensorimotor dysfunction could potentially cause sensory alteration and orofacial pain.^53^

Early studies examined somatosensory responses in teeth. In 1965, Scott and Tempel developed a technique to conduct electrophysiological studies in animal teeth by placing two metal electrodes (within few millimeters) in direct contact with exposed dentin through cavities prepared in cats’ teeth.^54^ In 1972, Scott provided evidence that dentin is innervated.^55^ Later, the anatomical relationships between the odontoblasts, their processes, and the sensory nerve endings were described in human teeth by studying sensory responses to thermal stimulation.^56^ Others demonstrated that dental pain can originate from the activation of nociceptive receptors at the pulp‐dentin junction.^57^, ^58^, ^59^, ^60^, ^61^

Olgart showed that local application of stimuli such as cold and heat could produce nerve impulses that were associated the with perceived pain sensations verbally expressed by the subject. We now know that alterations in pulpal blood flow as a result of aging or disease can affect these somatosensory findings.^8^ Edwall and Scott were one of the first groups to show that reductions of pulpal blood flow can increase excitability and evoke action potentials in the nerves innervating teeth.^62^

### 2.7 Measurements of circulating blood flow and temperature

Understanding the complex pathophysiology affecting circulating blood flow is also important for accurate pulpal diagnosis. Previous studies have investigated methods such as pulse oximetry, Laser Doppler flowmetry (LDF), and crown surface temperature change as ways of measuring pulpal blood flow, and each are described below in detail. However, none of these methods have been established as a standard of care in clinical dentistry.^63^, ^64^

#### 2.7.1 Pulse oximetry

Earlier studies suggested that blood oxygenation within teeth could be measured.^10^ Modern pulse oximeters now provide an inexpensive and efficient method to measure blood oxygenation. Pulse oximetry can detect oxygen saturation levels of the pulp tissue based on spectrophotometry using a light source (wavelengths 760 and 850 nm).^65^, ^66^, ^67^, ^68^ In one of the first studies demonstrating the utility of pulse oximetry in dentistry, Schnettler and Wallace showed that a pulse oximeter could detect pulse rate and oxygen saturation for vital teeth and not for root canal treated teeth. This work has now been replicated by several groups.^20^, ^65^, ^66^, ^67^, ^69^ Interestingly, Gopikishna showed that normal oxygen saturation in human permanent teeth was lower than systemic blood measured in fingers, 75% to 85% versus 98%, respectively.^70^ The sensitivity of the pulse oximeter to detect pulp vitality was 100% in comparison to sensitivity of cold (81%) and EPT (71%) methods. Unlike sensitivity, the specificity for pulse oximetry testing was very similar to cold and less compared to EPT testing for detecting pulp vitality in both mature and immature permanent central incisors.^71^ One of the limitations of using a pulse oximeter in dentistry is the differences in the optical properties of the teeth as the infrared light deflects through the teeth to the photodetector. The infrared light could also scatter due to its close proximity to surrounding gingival tissue.^72^, ^73^

#### 2.7.2 LDF

LDF is another method to measure blood flow using light (Helium Neon 632.8 nm). LDF works on Doppler principle – light undergoes a frequency shift and scatters as it passes through moving red blood cells.^74^ The light is scattered back onto photodetectors to measure pulpal blood flow.^75^, ^76^ LDF was originally designed for the measurement of organs that have abundant blood flow, such as the brain and skin, not for the dental pulp due to its low blood volume and/or low blood flow velocity.^77^ Prevoius studies noted many challenges in using LDF, especiallly when it is used to measure blood flow in the soft tissue of the oral cavity. LDF is spatially limited (1 mm^3^), and the unknown morphology of the vasculature affects probe placement and subsequently the accuracy of LDF output. LDF outputs are also negatively impacted by head movement of the patient and movement of the hand‐held probe by the operator.^78^ Contamination of signals from backscatered light on surrounding tissue and/or interference of ambient light together lead to non‐linear output in LDF measures that are difficult to interpret.^20^

#### 2.7.3 Temperature

Tooth temperature has also been proposed as measure of determine pulp vitality. For instance, Fanibunda et al. reported that vital teeth are warmer at rest and unlike non‐vital teeth they can rewarm more quickly when cooled down.^79^, ^80^, ^81^, ^82^ Other studies showed that despite having the same temperature at rest for vital and nonvital teeth, there is a delay in regaining heat in nonvital teeth versus vital teeth.^83^ One of the limiting factors in incorporating temperature measurements in clinical practice is that there is still no study to date that suggests a relationship between temperature and the degree of pulpal inflammation.^84^

Despite these limited data, noninvasive physiological monitoring techniques such as pulse oximetry, LDF, and crown surface temperature change may provide objective measures of pulp status that could be beneficial for clinical pulp vitality diagnosis.

## 3 QST IN OROFACIAL PAIN AND DENTISTRY

QST refers to a set of non‐invasive procedures for assessing sensory function. QST procedures are psychophysical in nature, and involve application of objective, quantifiable physical stimuli that evoke behavioral (e.g., verbal) responses from the individual being tested. QST has been used for decades in clinical research for cutaneous and mucosal assessment of pain sensitivity.^32^, ^85^, ^86^, ^87^, ^88^, ^89^, ^90^ It can also be used for patient subgroup classification and prognosis.^91^ Lastly, QST can help make inferences about the underlying mechanisms of pain and improve diagnostic accuracy in orofacial pain patients. For example, trigeminal neuropathic pain can be more accurately and reliably diagnosed using QST of innocuous mechanical and thermal stimulation.^36^ In many cases, patients with peripheral nerve damage present with hypoesthesia to warm and sometimes cool stimuli.^92^ TMD patients often experience hyperalgesia on palpation and/or thermal stimulation. This hyperalgesia is present in the orofacial region and also at remote, asymptomatic sites, like the forearm.^9^, ^17^, ^18^, ^87^, ^93^, ^94^, ^95^, ^96^ Evidence also supports perceptual amplification of nonpainful auditory tones in TMD patients.^97^ On the other hand, QST has revealed a reduced sensitivity to innocuous vibrations applied to the cheek (and to a lesser extent to the arm), compared to healthy participants, suggesting that ongoing pain signals may be “gating” (or masking) innocuous somatosensory inputs.^97^ The hyperalgesia observed in TMD may result from increased pain‐excitatory processes in the CNS (e.g., measured as increased temporal summation of pain) and/or impaired endogenous inhibition (e.g., measured as reduced conditioned pain modulation).^98^

Dentists have long used externally applied stimuli to determine a tooth's vitality as a form of “bedside” QST.^49^ The most commonly used tests stimulate the pulp by means thermal stimuli (e.g., cold testing) or electrical current (e.g., Electrical Pulp Test –EPT). These tests are used routinely in clinical practice to evaluate pulpal status, including the presence of healthy, inflamed, or necrotic pulp.^49^, ^99^, ^100^ However, a significant limitation in the current clinical implementation of these techniques in the assessment of dental pain is that perceptual responses are measured only in the form of qualitative and dichotomous “yes / no” subjective reports of the patient's evoked sensory experience.

Thermal testing includes assessment of tooth sensitivity to temperature changes via application of cold and heat stimuli. In this test, patients report the sensation they perceive during stimulus application. Overall, reports of pain sensations without lingering pain indicate healthy pulp, with lingering pain suggest inflamed pulp, and finally reports of no sensation may indicate necrotic pulp. According to the Brännström Hydrodynamic Theory, temperature changes cause dentinal fluids to rapidly move within the dentinal tubules which in turn induce activation of nociceptive A‐delta fibers within the pulp‐dentin complex.^70^ Application of heat, however, must be used with caution as it produces lingering pain through activation of C‐fibers pain and may increase pulp inflammation.^101^, ^102^ Cold application is safer as it does not produce detrimental effects on the pulp tissue.^103^, ^104^ Several methods of cold delivery are routinely used, such as ice stick (0°C), ethyl chloride (‐5°C), and dichlorodifluromethane (DDM). In clinical practice, it is more common to use DDM as it evokes a quicker response from the pulp in comparison to the other methods.^104^, ^105^, ^106^

EPT involves indirect electrical stimulation of the pulpal nerves through the tooth surface. In a series of early studies, Mumford used EPT for the diagnosis of pulp status by measuring the EPT – the lowest physical intensity of electrical stimulation that a person perceives to be just barely painful.^50^, ^107^ In theory, pulpal disease and its degenerative changes can lead to changes in pain threshold such that there are lower pain thresholds (increased pain sensitivity) in acute pulpitis and higher pain thresholds (decreased pain sensitivity) in chronic pulpitis. However, in clinical practice, Mumford's study sensations produced during EPT varied substantially among patients and this measure was not useful for diagnostic purposes. According to Mumford, EPT is a reliable test for identification of vital pulps, but not necessarily a reliable test for classifying pulp pathology using measures of pain sensitivity.^50^, ^107^

## 4 NONCOMMUNICATIVE PATIENTS AND CHALLENGES IN DENTAL PAIN ASSESSMENTS

Diagnosis and treatment of dental pain in young children, as well as in children or adults with CI, is very challenging given the current use of subjective measures for clinical pain assessments. Individuals with CI vary greatly in their experience of pain, and in their response to painful interventions based on their cognitive and emotional maturity.^108^ Dental care providers need to acknowledge these variations and be willing to understand the unique presentation of pain in these unique populations whether it is conveyed by verbal self‐report or only in behavioral cues such alternations in their feeding, sleeping and routine activities of daily living.

Self‐report of pain is often not reliable and sometimes not available in non‐verbal/non‐communicative individuals. In these cases, the history of pain relies heavily on remarks reported from a third party, e.g., parents, caregivers, teachers, etc. It is important to acknowledge observations provided by these caregivers as they know the patients very well and in different environments outside the clinic. They can also differentiate any subtle changes in behavior and notice cues outside the individuals’ normative behavior that may suggest the presence of pain.

When treating pediatric patients, it appears that cognitively impaired children experience pain more significantly in comparison to cognitively intact children.^109^, ^110^ Unfortunately, because of their limited verbal communication, pain in children with CI may be undertreated.^111^ Therefore, assessment and localization of dental pain is a complicated process when treating these children. Inadequate diagnosis of pain can lead to poor pain management; and unrelieved pain can have a negative physical and cognitive impact on the overall well‐being of these children. Given these challenges, the assessment of pain and pulp status in these populations may benefit most from the use of objective diagnostic methods.

## 5 BRIDGING THE GAP BETWEEN QST AND PULP VITALITY TESTING TO IMPROVE CLINICAL CARE

Dentists frequently struggle to answer questions such as “Is the pain coming from the tooth or another structure?” and “Is the tooth alive or dead?” These are critical questions in a wide variety of individuals presenting with acute and chronic orofacial pain. We contend that in order to better diagnose pulp vitality and standardize diagnoses across clinical providers, research should focus on improving our diagnostic tools to become more objective and quantifiable. For example, we propose that combining “research‐grade” QST methods that use well‐controlled, quantifiable stimuli with objective physiological readouts (e.g., pulse oximetry, LDF, temperature change) will significantly improve the likelihood of early diagnosis and treatment of dental pulp pathology, before irreversible damage occurs. Technology such as this would be of particular benefit in noncommunicative individuals and those with CI. However, as described earlier, each of these methods, as currently practiced, has limitations and challenges that need to be addressed prior to widespread clinical adoption.

## 6 CONCLUSION

The assessment of orofacial pain and pulp vitality is challenging, especially in nonverbal and impaired populations. Dentists regularly employ a variety of self‐report and sensory techniques in the clinic to aid in the diagnosis and treatment of tooth‐related disease. This review discusses nociceptive, neuropathic, and centralized (CNS) mechanisms that underlie acute and chronic dental pain. It details the measurement of somatosensory responses and pulpal blood flow as objective measures of tooth health and pain. It also introduces the measurement stimulus‐evoked sensations (i.e., QST) as practiced in research settings and compares it with existing qualitative and dichotomous “yes / no” aspects of sensory testing currently practiced at the point of care. Finally, it proposes that bridging these varied methodologies will significantly improve diagnosis and treatment of orofacial pain and pathology. It is critical the field of dentistry progresses toward a mechanistic understanding of pain to better identify individuals who are at risk of failing to respond to our interventions and to limit procedures to only those who will benefit from them.

## CONFLICT OF INTEREST

Each author completed a separate conflict of interest form from Wiley's website and included in the uploaded files on this submission. None of the authors has any conflict of interest regarding the publication of this manuscript.
